# Comparative Assessment of Silver Nanocomposites’ Biological Effects on the Natural and Synthetic Matrix

**DOI:** 10.3390/ijms222413257

**Published:** 2021-12-09

**Authors:** Mikhail A. Novikov, Eugeniy A. Titov, Larisa M. Sosedova, Viktor S. Rukavishnikov, Vera A. Vokina, Oleg L. Lakhman

**Affiliations:** East Siberian Institute of Medical and Ecological Research, 665827 Angarsk, Russia; g57097@yandex.ru (E.A.T.); sosedlar@mail.ru (L.M.S.); rvs_2010@mail.ru (V.S.R.); vokina.vera@gmail.com (V.A.V.); lakhman_o_l@mail.ru (O.L.L.)

**Keywords:** nanocomposites, nanosilver, brain, rats, arabinogalactan, poly-1-vinyl-1,2,4-triazole, histology, immunohistochemistry

## Abstract

The aim of our investigation was to make a comparative assessment of the biological effects of silver nanoparticles encapsulated in a natural and synthetic polymer matrix. We carried out a comparative assessment of the biological effect of silver nanocomposites on natural (arabinogalactan) and synthetic (poly-1-vinyl-1,2,4-triazole) matrices. We used 144 three-month-old white outbred male rats, which were divided into six groups. Substances were administered orally for 9 days at a dose 500 μg/kg. Twelve rats from each group were withdrawn from the experiment immediately after nine days of exposure (early period), and the remaining 12 rats were withdrawn from the experiment 6 months after the end of the nine-day exposure (long-term period). We investigated the parietal–temporal area of the cerebral cortex using histological (morphological assessments of nervous tissue), electron microscopic (calculation of mitochondrial areas and assessment of the quality of the cell nucleus), and immunohistochemical methods (study of the expression of proteins regulating apoptosis bcl-2 and caspase 3). We found that the effect of the nanocomposite on the arabinogalactan matrix causes a disturbance in the nervous tissue structure, an increase in the area of mitochondria, a disturbance of the structure of nerve cells, and activation of the process of apoptosis.

## 1. Introduction

Technical progress in the global nanoindustry is aimed at creating new, highly effective diagnostic and unique therapeutic nanoscale agents. This is possible due to the biospecific properties of nanoparticles “attached” to polymers designed for specific delivery and binding of nanoparticles to biological targets. Realizing their physicochemical and biological effects will raise the degree of solutions of most diagnostic and therapeutic problems to a new level [1,2,3]. Composite materials containing silver nanoparticles have unique properties and are promising for medicine. At the same time, nanosilver retains its inherent universal aseptic properties of silver macroforms. It can exert a specific effect at a minimum dose, which makes it possible to reduce the cost of silver-based drugs and make them available for the treatment of many infectious diseases [4,5,6,7]. The nano-stabilizing efficiency of the matrix has great importance in the formation of silver nanocomposites. The Irkutsk Institute of Chemistry SB RAS synthesized nanobiocomposites on natural (arabinogalactan (AG)) [8] and synthetic (poly-1-vinyl-1,2,4-triazole (PVT) matrices [9]). Nanobiocomposites are biocompatible, highly coordinating, and soluble [10,11,12]. However, the use of these nanocomposites is impossible without preliminary safety studies. First of all, research should study the patterns of their impact on health at the cellular and subcellular levels. In the scientific literature, there is an insufficient number of criteria for assessing the toxic effect of nanocomposites. Prospects for the widespread introduction of nanocomposites containing nanosilver [13] require a timely and in-depth study of their biological effects, including remote ones. A significant number of researchers have evaluated the biological effects of silver nanoparticles in preclinical studies, mainly using acute or subchronic experiments [14,15,16,17], while the possibility of long-term exposure to nanoparticles has not been studied.

The aim of our investigation was to compare the biological effects of silver nanoparticles encapsulated in a natural and synthetic polymer matrix.

## 2. Results

### 2.1. Histological Results

**Figure 1:**
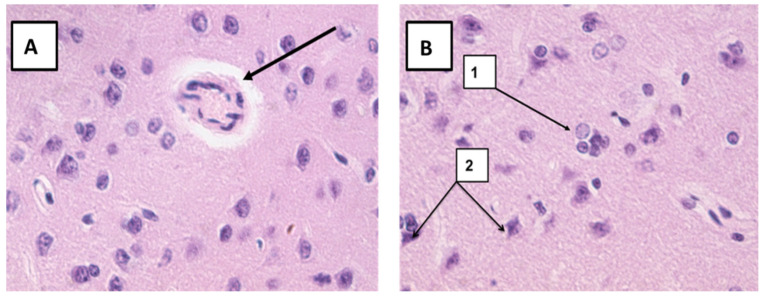
Microphoto of a section in cerebral cortex nAG exposed rats (early period). (**A**)—Expansion of the perivascular spaces, the artery wall is thickened, loose, and myocytes and endothelial cells are swollen (arrow); (**B**)-1—neuronophagy, 2—dark neurons. Hematoxylin-eosin. Mag. ×400.

**Figure 2:**
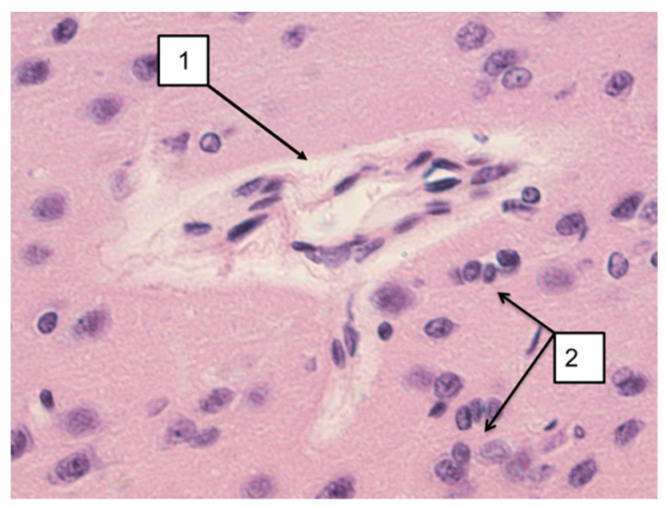
Microphoto of a section in cerebral cortex nAG-exposed rats (long-term period). 1—Expansion of the perivascular spaces, 2—neuronophagy. Hematoxylin-eosin. Mag. × 400.

In the nAG group, disturbances in the structure of the nervous tissue were revealed: expansion of the perivascular spaces, swelling of vascular bundles, neuronophagy, swelling of myocytes and vascular endotheliocytes, and dark neurons (Figure 1). These disorders were also recorded in the long-term period (Figure 2).

**Figure 3:**
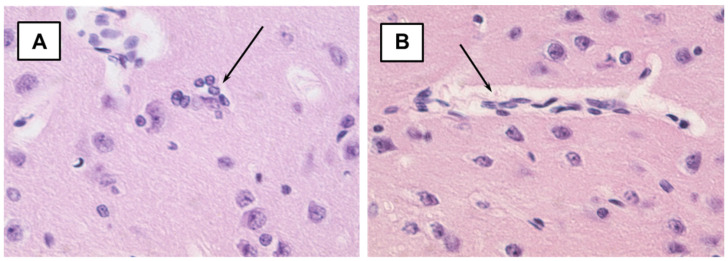
Microphoto of a section in cerebral cortex AG-exposed rats. (**A**)—Early period. Expansion of the perivascular spaces (arrow); (**B**)—Long-term period. Neuronophagy (arrow). Hematoxylin-eosin. Mag. × 400.

In animals of the AG group, at all examination periods, a slight expansion of the perivascular spaces and neuronophagy was revealed, which indicates metabolic changes in the structure of cells and tissues (Figure 3).

**Figure 4:**
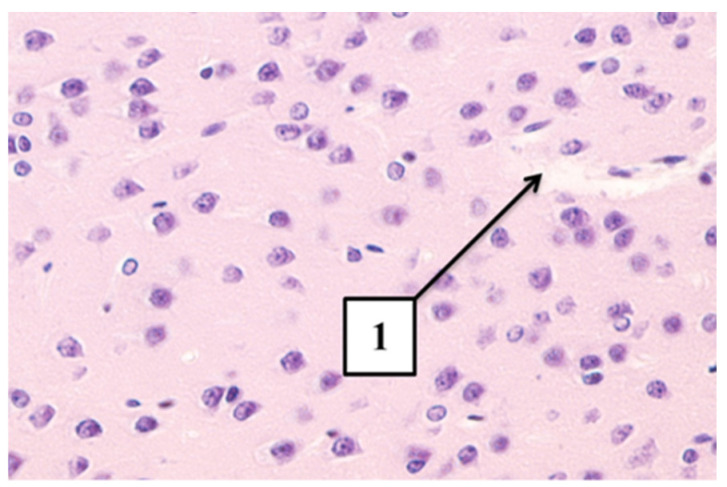
Microphoto of a section in cerebral cortex nPVT-exposed rats (early period). 1—swelling of the conductive fibers. Hematoxylin-eosin. Mag. × 400.

**Figure 5:**
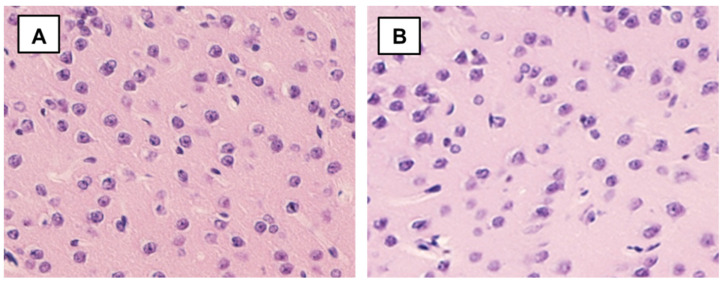
Microphoto of a section in cerebral cortex CS-exposed rats. (**A**)—Early period; (**B**)—Long-term period. All indicators are normal. Hematoxylin-eosin. Mag. × 400.

Morphological examination of brain tissue preparations from exposed nPVT rats showed only insignificant swelling of the conductive fibers of the animals in the early period of investigation (Figure 4). In the PVT group only, single changes were observed that did not have multiple confirmations and did not differ from the control. In rats treated with CS, the results of the morphological examination of brain preparations were comparable with the results of the control group (Figure 5).

### 2.2. Results of Ultrastructural Analysis

**Figure 6:**
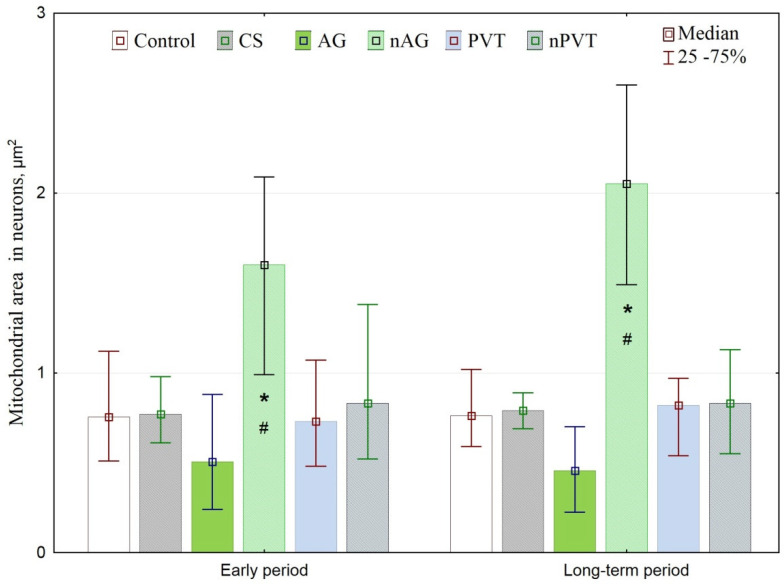
Mitochondrial area (μm^2^) in neurons, Med (Q25–Q75). Note: the differences are statistically significant at *p* < 0.01, *—compared with the control group, ^#^—compared with the AG group.

Ultrastructural examination of the mitochondrial area in the early and long-term periods revealed a significant increase in the neuronal mitochondria analysis of neurons in the nAG group in all periods. CS, AG, PVT, and nPVT groups did not reveal an increase in the area of mitochondria, which indicates the preservation of the metabolic processes in neurons (Figure 6). 

**Figure 7:**
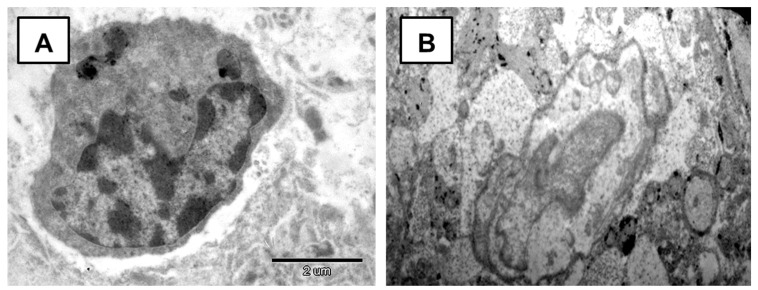
Electronogram of nucleus of cortical neurons. (**A**)—Deformation of the nucleus in early period. (**B**)—karyorrhexis in long-term period. Mag. × 12,000.

Ultrastructural analysis of the brain tissue of rats exposed to nAG revealed an increasing dynamic deformation of the neuron nucleus (Figure 7). An irregular, deformed shape and an increase in the area of mitochondria indicate an unfavorable effect of silver nanoparticles on intracellular structures and are indirect evidence of the ability of nanosilver to penetrate from the polymer matrix into the brain.

### 2.3. Results of the Immunohistochemical Investigation

**Figure 8:**
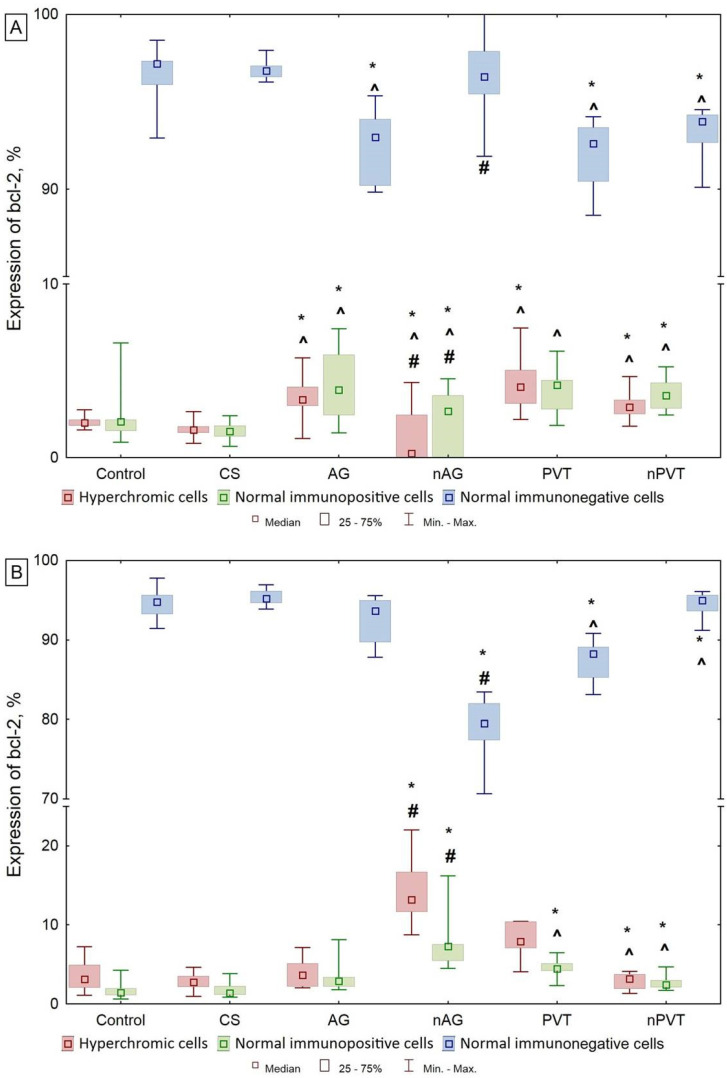
Expression of bcl-2, % of the total number of cells (Med (Q25–Q75)). (**A**)—Early period; (**B**)—Long-term period. Note: *—the differences are statistically significant compared with the control group at *p* < 0.01; ^#^—the differences are statistically significant compared to the AG group at *p* < 0.01; ^^^—the differences are statistically significant compared with the CS group at *p* < 0.01.

Analysis of the activity of regulatory proteins in neurons indicated an increase in the expression of anti- and pro-apoptotic proteins in neurons only in the case of the injection of the nanocomposite with the AG matrix. The study of the expression of the apoptosis-inhibiting protein factor bcl-2 showed that there was a statistically significant increase in the percentage of hyperchromic neurons in the nAG group in comparison with the control, CS, and AG groups in the early period of the examination (Figure 8). Simultaneously, there was a significant increase in the number of normal immunopositive cells and, accordingly, a decrease in normal immunonegative cells. The results obtained indicate the activation of the expression of the apoptosis-inhibiting protein factor and the mobilization of defense mechanisms that prevent the development of apoptosis. In the long-term period, the revealed direction of the changes remained. At the same time, hyperchromic and normal cells immunopositive to bcl-2 with a simultaneous reduction in the number of normal cells without expression to the protein under study were detected much more often than the control and AG groups.

**Figure 9:**
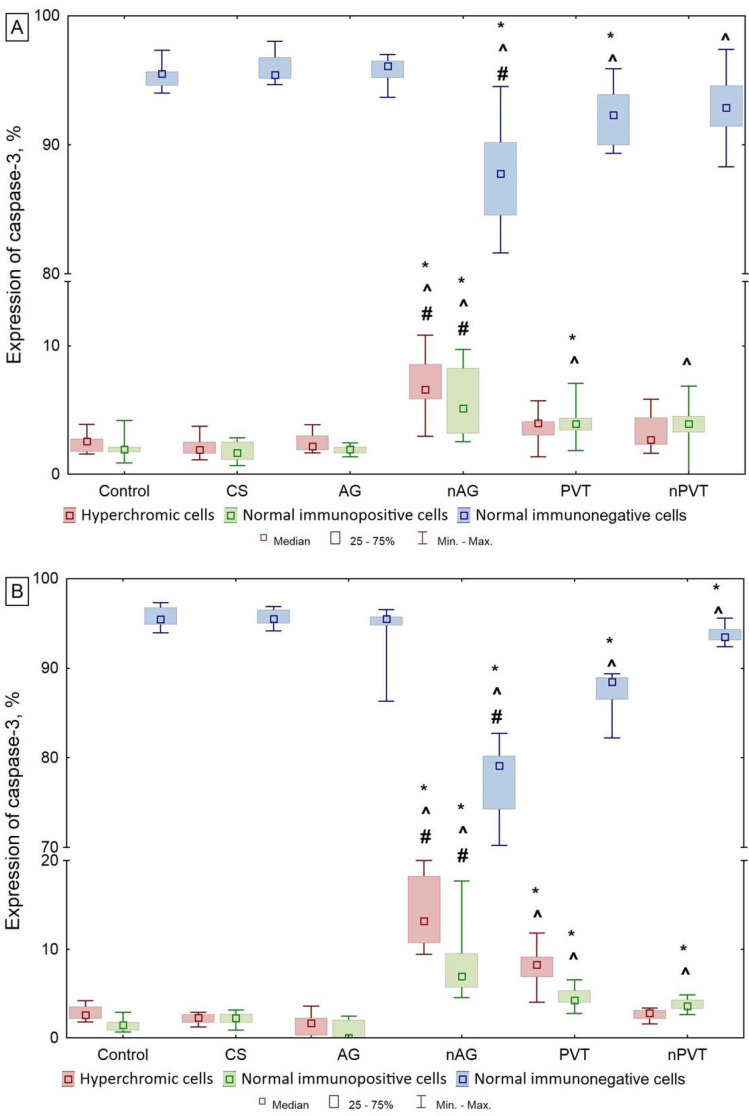
Expression of caspase 3, % of the total number of cells (Med (Q25–Q75)). (**A**)—Early period; (**B**)—Long-term period. Note: *—the differences are statistically significant compared with the control group at *p* < 0.01; ^#^—the differences are statistically significant compared to the AG group at *p* < 0.01; ^^^—the differences are statistically significant compared with the CS group at *p* < 0.01.

The study of caspase-3 expression revealed statistically significant differences between the nAG and AG groups in both periods (Figure 9). There was a reduction per unit area of the number of normal unchanged cells without expression of the pro-apoptotic protein caspase-3. In contrast, hyperchromic cells and normal cells expressing caspase-3 increased significantly. The revealed results indicate the activation of apoptotic processes immediately after the end of the exposure of the nanobiocomposite. This is consistent with the data on the expression of the apoptosis inhibitor bcl-2, which begins to exert a protective effect simultaneously in response to the activation of the apoptotic process under the influence of nAG. In the long-term period of examination, the number of hyperchromic and normal cells expressing the caspase-3 protein becomes even higher, which indicates an increase in the process of apoptosis.

Comparing the expression indices of the two investigated regulatory proteins, it was found that, in the nAG group, at an early stage of the examination, when evaluating the expression of the caspase-3 protein, the number of hyperchromic cells was 1.37 times higher than when evaluating the expression of the bcl-2 protein. The number of normal immunopositive cells expressing bcl-2 increased slightly compared to the AG group, while the number of the same cells expressing caspase-3 was 2.65 times higher. In the long-term follow-up, in the nAG group, the number of hyperchromic and normal immunopositive cells with caspase-3 expression also significantly exceeds the analogous indicators of bcl-2 protein expression (4.5 and 4.4 times, respectively), which indicates a continuous active apoptotic process that suppresses the action of an anti-apoptotic protein. An analysis of the results of the expression of apoptosis-regulating proteins in rats exposed to nPVT did not reveal significant changes in comparison with the introduction of a pure polymeric PVT matrix, indicating the activation of apoptosis in nerve cells throughout the entire observation period.

## 3. Discussion

Numerous studies have put an end to the discussion about the possibility of metal nanoparticles penetrating the blood–brain barrier, which restricts the flow of many substrates into the brain. Experimental studies have established alternative changes in the blood–brain barrier and brain tissue with different parenteral routes of entry of metal nanoparticles (Ag, Cu, Al, etc.) [18,19,20,21,22]. The deformation of the nuclei of neurons and an increase in the mitochondrial area revealed in this study, along with disturbances in the structure of the nervous tissue of the cerebral cortex, increased in the long term after exposure to nAG and may have a significant effect on the processes of intracellular metabolism. We recently showed that exposure to gadolinium nanoparticles encapsulated in a polymer matrix of AG leads to an increase in the number of degeneratively altered neurons and neuronophagy in the sensorimotor cortex in rats [23]. In addition, we established the selective cytotoxicity of copper oxide nanoparticles on the AG matrix, causing a decrease in astroglial cells, which can also lead to disruption of the normal homeostasis of the nervous tissue [24]. It is believed that the biological effects of metal nanoparticles can be mediated either by the direct action of nanoscale structures entering the tissues as such or by the influence of ions that can be separated from the surface of the introduced nanostructures [25]. At the same time, it is known that one of the main mechanisms of action of how nanoparticles damage the cell is the generation of radical forms of oxygen and the induction of oxidative damage to DNA in the brain tissue [26,27].

A comparison of the results of a morphological study of the nervous tissue with the data on the expression of caspase-3 and bcl-2 proteins allows us to conclude that nanosilver encapsulated in a polymer matrix arabinogalactan is capable of inducing an apoptotic cascade in neurons of the cerebral cortex, which, after nine-fold administration of the nanobiocomposite, is located on the initial stage of dysregulation of the mechanisms of programmed cell death and gradually, over time, leads to a state of the cell with characteristic signs of an active apoptotic process. Taking into account that the number of hyperchromic cells increases with the introduction of nAG in the long-term, it can be concluded that cell death occurs with the start of the apoptosis program and is caused by other mechanisms of cell damage and death. In our opinion, when programmed cell death is triggered, the mitochondrial pathway of cell entry into apoptosis is quite probable when active caspases formed from procaspases suppress the activity of the anti-apoptotic protein bcl-2. Caspase 3 is one of the end points of the cascade of activation of proteolytic enzymes leading to programmed cell death [28] and the formation of intracellular defense mechanisms.

The activity of the apoptosis process has its own characteristics depending on the type of metal nanoparticles. In recent studies, we did not reveal changes in the expression of caspase-3 and bcl-2 under the influence of iron oxide nanoparticles encapsulated in AG, while a decrease in the total number of neurons in the tissue of the sensorimotor cortex was observed, which makes it possible in this case to exclude the effect of apoptosis mechanisms on neuronal death [29]. Gadolinium nanoparticles on the AG matrix at a similar dose caused a decrease in the expression of bcl-2 in the rat brain [23], which is consistent with the results of Alarifi S. et al. (2017) on the suppression of the expression of Bcl-2 mRNA when exposed to nanoparticles of gadolinium oxide Gd_2_O_3_ on the culture of human neuroblastoma cells [30]. A decrease in the activity of this protein makes the cell more susceptible to apoptosis. At the same time, when exposed to Gd_2_O_3_ nanoparticles, an increase in the activity of the Bax protein, capable of activating the apoptotic process, was established [30]. The induction of apoptosis and activation of P53-dependent signaling in neurons under the influence of titanium dioxide (TiO_2_) nanoparticles were revealed [31]. In neuronal stem cells of the CNS, apoptosis can also be caused by zinc oxide nanoparticles [32]. One of the reports showed the role of alumina nanoparticles in development-induced apoptosis against the background of deterioration in the skills of spatial orientation in animals in a maze, which confirms the key role of nanoaluminum in neurotoxic reactions [33].

In our opinion, the appearance of long-term effects of action upon administration of silver nanobiocomposite to rats is due to the long-term persistence of nanoparticles in the body with their insignificant elimination from the body, the ability to accumulate material, and the formation of conglomerates in cell structures and intercellular space. The results obtained gave grounds to conclude that there was no difference in the biological effects of the nanobiocomposite containing nanosilver in the synthetic HTP matrix and the “pure” PVT on rats. PVT and its derivatives, due to the peculiarities of their chemical structure (absence of open chemical bonds, general chemical stability), do not disintegrate into individual components, do not integrate into the chain of biological reactions in the body, and are excreted practically unchanged. We assume that, due to the closed chemical structure, nanosilver does not release from PVT-nanocomposite, does not penetrate the blood–brain barrier, and does not take part in the reactions of cellular metabolism.

## 4. Materials and Methods

### 4.1. Silver Nanocomposites’ Preparation and Characterization

All the chemicals were from Favorsky Institute of Chemistry SB RAS (Irkutsk, Russia). Before the introduction, all substances were suspended in distilled water to prepare an initial suspension (1 mg/mL). Nanocomposite nAG contains silver nanoparticles in the zero-valent state of a spherical shape with size 4–8 nm; silver content was 3.1% [34]. Arabinogalactan is a water-soluble white or creamy powder, tasteless and odorless with a patented production technology [8]. The macromolecule of arabinogalactan is represented by the residues of galactose and arabinose. 

Nanocomposite nPVT contains spherically shaped silver nanoparticles with sizes 2–6 nm; the silver content in sample was 7.03% [35]. Synthetic polymer PVT is a water-soluble biocompatible polymer with chemical resistance and thermal stability, capable of stabilizing particles of silver nanoparticles in the zero-valent state [9].

All chemicals was dissolved in distilled water, working solutions were prepared on the day of administration.

### 4.2. Animals and Experimental Design

One hundred forty-four three-month-old white outbred male rats (weight 180–200 g) were used for the investigation. The animals were randomly assigned to six groups (*n* = 24): two groups were exposed to silver nanoparticles encapsulated in natural biopolymer arabinogalactan (group nAG) and synthetic poly-1-vinyl-1,2,4-triazole ( group nPVT) at a dose 500 μg/kg. This dose was chosen based on the results of previous investigations and was 1/10 of LD_50_. Two groups received an aqueous solution of polymers without nanoparticles (groups AG and PVT) in an equivalent volume. Animals of CS group received an aqueous dispersion of colloidal silver, stabilized by casein, with a silver content of 8%. Animals of control group received distilled water. Solutions were administered orally using an atraumatic probe for 9 days. 

The investigation was carried out in 2 stages: 12 rats from each group were withdrawn from the experiment immediately after exposure (early period), 12 rats—6 months after the end of exposure (long-term period). The examination included morphological studies of the nerve tissue of the temporoparietal zone of the cerebral cortex, electron microscopy of neurons in the cerebral cortex, and determination of the activity of the proteins caspase 3 and bsl-2.

All animals were kept under 12/12 h light/dark cycle, on a ventilated shelf, and under controlled temperature and humidity conditions (22–25 °C and 55–60% humidity). Experimental animals were obtained from the vivarium of Federal State Budgetary Scientific Institution “East Siberian Institute of Medical and Ecological Research” (FSBSI ESIMER) and kept on a standard diet (BioPro Russia, water ad libitum). 

All animal experiments were approved by the ethical committee of FSBSI East-Siberian Institute of Medical and Ecological Research (identification code: E05/20; date of approval: 4 March 2020, amended/approvals every 6 months) and carried out in compliance with the rules of humane treatment of animals in accordance with the requirements of the International Recommendations for Biomedical Research Using Animals (WHO, Geneva, 1985), U.K. Animals (Scientific Procedures) Act (UK, 1986) and National Institutes of Health guide for the care and use of laboratory animals (NIH Publications No. 8023, revised 1978). 

### 4.3. Histological Investigation

To perform morphological studies of the nervous tissue, the animals underwent euthanasia by decapitation. The brain from each animal under study was removed and fixed in neutral buffered formalin solution (10%), dehydrated with ascending concentrations of ethanol (70, 80, 90, 95, and 100%), and placed in a homogenized paraffin medium for histological studies HistoMix (BioVitrum, Russia). Then, using an HM 400 microtome (Microm, Germany), serial horizontal sections with a thickness of 4–5 μm were made at Bregma—6.10 mm level, Interaural—3.90 mm, which were stained on ordinary histological slides with hematoxylin-eosin for observation microscopy [36].

### 4.4. Electron Microscopy Investigation

Electron microscopy was used for ultrastructural assessment of the state of neurons in the cerebral cortex. The studies were carried out using a Leo 906E electron microscope (Zeiss, Germany). The number and cross-sectional area of mitochondria and state of neuronal nuclei were determined at different periods of the investigation.

### 4.5. Immunohistochemical Investigation

An immunohistochemical method was used to determine the activity of caspase 3 and bsl-2 proteins. Sections obtained on a microtome were placed on poly-L-lysine coated slides (Menzel-Gläser, Braunschweig, Germany) and stained for antibodies to the caspase-3 protein and antibodies to the bsl-2 protein (Monosan, Uden, Netherlands) in accordance with the protocol proposed by the manufacturer. Visualization of stained and fixed micropreparations was carried out using a light-optical research microscope Olympus BX 51 (Olympus, Tokio, Japan) with microimages input into a computer using an Olympus camera. The analysis of the obtained photographic materials was carried out using the Image Scope S system (SMA, Moscow, Russia). The following analysis parameters were selected: number of immunopositive and immunonegative normal neurons and hyperchromic neurons. Cells stained for antibodies to caspase-3 and bsl-2 proteins were immunopositive, and unstained cells were immunonegative. Cells without a well-defined nucleus were considered hyperchromic, which is a sign of damage. The number of cells was determined per unit area of the histological preparation (0.2 mm^2^).

### 4.6. Statistical Analyses

Statistical analysis of the research results was carried out using the Statistica 6.1 software package (Statsoft, Tulsa, OK, USA). The Shapiro–Wilk W-test was used to decide the type of feature distribution. To compare groups, we used the Mann–Whitney U-test. Null hypotheses about the absence of differences between the groups were rejected at the achieved significance level of *p* ≤ 0.05.

## 5. Conclusions

A comparative analysis of the biological effects of silver nanoparticles encapsulated in various stabilizing matrices revealed the features of their effect. Pathological abnormalities expressed in the structure of the temporoparietal zone of the sensorimotor cortex of the rat brain, increasing over time, were found in the nanobiocomposite of silver nanoparticles and the natural polysaccharide arabinogalactan. At the same time, the introduction in a similar mode and dose of silver nanoparticles encapsulated in a synthetic matrix of poly-1-vinyl-1,2,4-triazole did not lead to any noticeable changes in the studied parameters early on or in the long-run. Features of the biological effects of silver nanoparticles encapsulated on various matrices can be used in medical research to reduce the adverse effects of silver nanoparticles.

## Data Availability

The data presented in this study are available from the corresponding author upon request.
